# Family Experiences, Needs, and Perceptions in Home-Based Hospice Care for Patients With Terminal Cancer: Meta-Synthesis and Systematic Review

**DOI:** 10.2196/71596

**Published:** 2025-06-19

**Authors:** Xin Ming Deng, Kanokwan Hounsri, Violeta Lopez, Wilson Wai-San Tam

**Affiliations:** 1 Alice Lee Centre for Nursing Studies, Yong Loo Lin School of Medicine, National University of Singapore, 10 Medical Dr, Singapore, 117597, Singapore, 65 90505570; 2 School of Nursing, Midwifery and Social Sciences, Central Queensland University, Rockhampton, Australia; 3 Department of Nursing, School of Nursing and Allied Medical Sciences, Holy Angel University, Angeles City, Philippines

**Keywords:** palliative, hospice, home care, cancer, meta-synthesis.

## Abstract

**Background:**

Home-based hospice care offers patients with terminal cancer the comfort of receiving care in a familiar environment while enabling family members to provide personalised support. Despite the critical role families play, the literature remains underexplored in terms of their experiences, needs, and perceptions. A robust qualitative synthesis is needed to inform improvements in palliative care services.

**Objective:**

This meta-synthesis aims to systematically review and synthesize qualitative evidence regarding the experiences, needs, and perceptions of family caregivers in home-based hospice care for patients with terminal cancer. The goal is identifying key themes that can improve caregiver support and service delivery.

**Methods:**

A systematic search was conducted across MEDLINE, Embase, Scopus, PsycINFO, CINAHL, Google Scholar, and relevant gray literature sources up to March 14, 2025. Studies were included if they focused on family caregivers’ experiences in home-based hospice care settings, excluding those that addressed only patients or health care providers. Two independent reviewers performed study selection, data extraction, and quality assessment using the Critical Appraisal Skills Programme checklist. Data were synthesized using a 3-step thematic synthesis approach, and the confidence in the findings was assessed via the GRADE-CERQual (Grading of Recommendations Assessment, Development, and Evaluation–Confidence in the Evidence from Reviews of Qualitative Research) framework.

**Results:**

Five studies published between 1989 and 2022 from diverse geographical regions (including Asia and Western settings) met the inclusion criteria. Two major themes emerged: (1) being physically and emotionally present, where caregivers expressed a strong commitment to remain with their loved ones, providing emotional support and maintaining a sense of control; and (2) sharing responsibilities, which underscored the importance of both formal support from palliative care teams and informal support from family and friends in mitigating caregiver burden. These findings directly address the study’s aims by illustrating how caregivers balance emotional commitment with the practical challenges of providing home-based care.

**Conclusions:**

Although family caregivers are dedicated to delivering high-quality, personalized care, they encounter significant emotional and logistical challenges. Variability in study settings, potential recall bias from retrospective interviews, and limited gray literature access may affect the generalizability of the findings. This meta-synthesis underscores the essential role of family involvement in home-based hospice care for patients with terminal cancer. The combined reliance on emotional commitment and shared responsibilities—with support from professional care teams—is vital for optimal care delivery. Future interventions should enhance formal and informal support systems to meet family caregivers’ diverse needs better.

## Introduction

### Background

Palliative care is an active, holistic approach aimed at relieving severe or chronic suffering and enhancing the quality of life for individuals with life-threatening illnesses at any stage of the illness trajectory, from diagnosis onward, whether curative treatments continue or not [1-3]. Hospice care, however, refers explicitly to palliative care provided when patients no longer pursue curative treatments, typically with a prognosis of 6 months or less, focusing on comfort, dignity, and quality of life during the end-of-life stages [2].

Cancer remains the second leading cause of death globally, with continually rising incidence rates each year [4]. Advances in cancer treatment and early detection have contributed to prolonged survival, even for patients in advanced stages. Nevertheless, extended survival often results in protracted suffering, posing significant physical, emotional, social, and spiritual challenges for both patients and their families, especially during the end-of-life phase [5]. Terminal cancer typically refers to patients with cancer with a prognosis of 6 months or less to live, at which point curative treatments are usually ceased and care transitions toward symptom management and comfort measures [2].

Home-based hospice care allows patients with terminal cancer to spend their final days at home, as many of them prefer [6,7]. This care typically comprises scheduled visits by health care professionals and 24-hour on-call support rather than continuous, around-the-clock in-home care [8]. Such services often include symptom management, holistic nursing care, and psychosocial and spiritual support tailored to individual family needs [9,10]. The popularity of home-based hospice care is increasing due to multiple factors, such as overcrowded hospital environments [11], rising complexity of symptoms and treatments [12], improvements in living standards and education [13], and a growing emphasis on maintaining quality of life at the end of life [14]. The aging population and a shift toward value-based care models have also contributed to the rising demand for home-based hospice services [15].

Family caregivers are pivotal in home-based hospice care, often providing daily care and managing multifaceted emotional and logistical responsibilities. In many cultural contexts, caregiving is perceived as a moral or filial obligation, significantly influenced by cultural norms and values that shape caregiver expectations and decision-making processes [16]. For example, in many Asian cultures, caregiving at home is deeply rooted in filial piety, emphasizing familial responsibility and moral duty toward elders [16,17]. Understanding caregivers’ culturally influenced experiences, attitudes, perceptions, and unique support needs is essential for effective and culturally competent interventions.

Given these considerations, home-based hospice care is experiencing increasing demand and attention, primarily due to its valuable support for family caregivers who assume multifaceted responsibilities involving intensive physical care, emotional support, and complex decision-making. These caregivers’ experiences, perceptions, and needs vary significantly, influenced by personal, cultural, and contextual factors. Therefore, understanding caregivers’ perspectives is essential for assessing the effectiveness of current hospice services and identifying opportunities for enhancing family support at home, ultimately leading to improved patient and caregiver experiences during this critical period.

### Objectives

The overall aim of this study was to update and synthesize qualitative research on home-based hospice care based on the experiences of family caregivers of patients with cancer.

The three specific objectives for this review were as follows:

1. To explore the experiences of families of patients with terminal cancer receiving home-based hospice care.
2. To examine attitudes and perceptions of families toward home-based hospice care.
3. To identify key needs within the context of home-based hospice care services.

This meta-synthesis seeks to address a significant gap in the current literature by conducting a comprehensive review of the experiences of family caregivers supporting patients with terminal cancer in home-based hospice care settings. By examining the caregiver experience, this study aims to assess whether existing palliative care provisions sufficiently meet their needs and provide insights for future improvements. Ultimately, this research will ensure that caregivers receive holistic and compassionate support during this critical phase of the illness trajectory.

## Methods

### Overview

The qualitative evidence from primary qualitative studies and mixed-methods studies were synthesized and integrated using the thematic synthesis method. The meta-synthesis protocol was reported following the PRISMA (Preferred Reporting Items for Systematic Reviews and Meta-Analyses) 2020 checklist [18] (Checklist 1). The meta-synthesis was guided by the 6 steps of qualitative research synthesis developed by Major and Savin-Baden [19], including formulating the questions, identifying studies, selecting studies and extracting data, appraising studies, synthesizing and finalizing data, and reflecting upon the process, which was based on the step-by-step qualitative research synthesis approaches by Noblit and Hare [20] and Sandelowski and Barroso [21]. The study protocol has been registered in PROSPERO (Prospective Register of Systematic Reviews) under registration number CRD42023486012.

### Eligibility Criteria

Identifying appropriate studies is crucial in alignment with step 2 of the Major and Savin-Baden [19] approach. Essential components in this identification process include (1) the scope of included studies, (2) inclusion and exclusion criteria, (3) quality assessment, (4) data synthesis method, and (5) criteria for reporting findings [22,23]. 

The criteria for considering studies for this review were based on the PICo (Population, Interest, Context) framework [24]. The inclusion criteria were as follows: (1) population: the studies involving adults who are family members of patients with terminal cancer; (2) interest: the experiences, attitudes, and needs regarding home-based hospice care services; (3) context: under the care of home-based hospice care service, particularly those with a physical home visit; and (4) the research design was qualitative or mixed methods. The exclusion criteria were as follows: (1) studies focusing on patients themselves, health care providers, or non–home-based hospice services (such as inpatient hospice or nursing home care), and studies involving only telemedicine visits, and (2) language is not English. The eligibility criteria are outlined in Multimedia Appendix 1.

### Information Sources

We searched 5 electronic databases—Scopus, Embase, MEDLINE, CINAHL, and PsycINFO—from inception until March 14, 2025. These databases were chosen for their relevance to qualitative research in various health care settings [25]. To maximize the range of articles retrieved, we searched Google Scholar and gray literature sources, including ProQuest, for unpublished dissertations. The complete search strategy can be found in Multimedia Appendix 2.

### Search Strategy

The search began by defining the scope of the study and addressing the research questions focusing on terminal cancer, home-based care, and palliative care. Broad search terms and synonyms were used to create a comprehensive search string encompassing all relevant keywords. The PICo framework [24] was used to guide search term generation. The full search strategies included an initial search of MEDLINE, Embase, Scopus, PsycINFO, and ProQuest up to September 13, 2023. Following our initial database search, we conducted an additional search in CINAHL on March 13, 2025, and Google Scholar on March 14, 2025, to ensure comprehensive coverage of relevant literature.

Controlled vocabulary terms were used for each database: Medical Subject Headings (MeSH) for PubMed and Emtree terms for Embase. Boolean operators and truncation symbols combined the terms according to each database’s specifications. This initial step helped us develop effective search strategies, become familiar with the terminology, and conduct preliminary searches. We then consulted a subject librarian to further refine these search terms and strategy. Subsequently, a formal literature search was conducted to identify and compile eligible studies, with language limited to English only.

### Selection Process

Adhering to the third step of the Major and Savin-Baden [19] meta-synthesis method, we used EndNote 21 (version 21.2.0.19537; Clarivate Plc) [26] to import articles and find duplicates. Subsequently, the titles and abstracts of the imported articles were screened for relevance using RAYYAN [27], using the blinding function to mitigate bias. This initial screening, followed by full-text screening, was independently conducted by 2 reviewers (XMD and KH), with discrepancies resolved through discussion to reach a consensus.

### Data Extraction

As the third step of Major and Savin-Baden’s [19] meta-synthesis approach, we meticulously extracted the qualitative data from the included articles. This process was conducted in 2 stages. First, a pilot test of the data-extraction form was performed by 2 reviewers (XMD and KH) and validated by the third reviewer (WWST) before extracting relevant information. Subsequently, the data-extraction form was applied, which included author, year of publication, study setting, aim, sample characteristics, methodology (population characteristics, sampling method, data collection, and data analysis), and key findings.

### Quality Assessment

For the quality appraisal, 2 investigators (XMD and KH) independently assessed each included study using the Critical Appraisal Skills Programme (CASP) checklist (2019) [28]. All discrepancies between the 2 investigators were resolved through discussion. The third reviewer (WWST) counterchecked the results to ensure accuracy and consistency. This assessment included statements of research aims, appropriate qualitative methodology, research designs, recruitment strategies, data collection, adequate relationship between researcher and participants, ethical issues consideration, the rigor of data analysis, statement of findings, and value of the study.

### Synthesis Methods

The data analysis used the 3-step thematic synthesis method [25] as the fifth step in the Major and Savin-Baden [19] approach. This method, based on Braun and Clarke’s thematic analysis techniques [29], was extensively used in nursing and medical research to identify intervention needs, appropriateness, acceptability, and factors influencing implementation. Thematic synthesis integrates findings from primary studies to identify prominent or recurrent themes within the relevant literature.

In the initial step of thematic synthesis, findings were extracted and coded line-by-line using Excel (Microsoft Corp). Reviewer XMD conducted the line-by-line coding, maintaining fidelity to the data and preserving the original concepts. Reviewer KH subsequently verified the codes to ensure alignment and completeness, facilitating identifying and categorizing key elements within the data.

The primary codes were then grouped based on conceptual similarities upon mutual agreement by reviewers (XMD and KH), resulting in a structured interpretation of the findings through the development of descriptive themes. These descriptive themes were subsequently synthesized into higher-level analytical themes. These analytical themes represented the key outcomes relevant to our meta-synthesis topic, achieved by merging and summarizing similar descriptive themes to highlight core insights and conclusions drawn from the data. The final process and results were screened thoroughly and confirmed by all reviewers (XMD, KH, WWST, and VL), with any disagreements resolved through consensus.

Third, the use of the approach by Major and Savin-Baden [19] involved 5 steps; the last was adopting the 3-step thematic synthesis method [25]. This nursing and medical method was used massively using Braun and Clarke’s thematic analysis methods [29] to determine intervention needs, appropriateness, acceptability, and factors regarding implementation.

### Confidence Measurement

After generating the analytical themes, a further quality appraisal stage using the GRADE-CERQual (Grading of Recommendations Assessment, Development, and Evaluation–Confidence in the Evidence from Reviews of Qualitative Research) approach [30] was used to evaluate the confidence level of our findings (Table 1). This aligned with the final step of Major and Savin-Baden’s [19] approach of “reflecting upon the process.”  

**Table 1.** Evidence profile table.

Major theme and summarized review finding		Methodological limitations	Coherence	Adequacy	Relevance	GRADE-CERQual^a^ assessment of confidence	References
**Being physically and emotionally present**							
	Belief that home palliative care provides better care than hospitals Commitment to care at home Cultural and moral obligations Personal reflections and challenges	No or very minor concerns	No or very minor concerns	Minor concern	Minor concerns	High confidence	[16,31-34]
**Sharing responsibilities**							
	Challenges in caregiving Formal support needs Informal support needs	No or very minor concerns	No or very minor concerns	No or very minor concerns	Minor concerns	High confidence	[16,31-34]

a GRADE-CERQual: Grading of Recommendations Assessment, Development, and Evaluation–Confidence in the Evidence from Reviews of Qualitative Research.

Two reviewers (XD and KH) conducted independent reviews and discussed discrepancies to reach a consensus. The GRADE-CERQual approach evaluates confidence based on 4 components: methodological limitations, coherence, adequacy of data, and relevance of included studies. Each element was categorized as having “no or very minor concerns,” “minor concerns,” “moderate concerns,” or “serious concerns,” leading to varying grades of confidence [35].

The assessment of methodological limitations aligns with the CASP appraisal, evaluating the trustworthiness of study findings by examining the appropriateness of the research methodology, which is closely related to the quality of the results [36]. Our assessment indicated that the findings were supported by articles with no to very minor concerns regarding methodological limitations.

The coherence assessment measures the relevance of the data from the included studies to the review findings [37]. Based on key findings extracted from the included studies, we confirmed that the data were relevant to the review findings (Table 2).

To assess the adequacy of the data, we evaluated both the quantity and richness of the data in relation to the review findings, in line with the GRADE-CERQual approach [38]. Studies with limited data, particularly those with findings supported by only 1 or 2 participant voices, were noted as having insufficient depth and quantity to robustly support specific review findings [31,39].

Lastly, we assessed relevance by evaluating the extent to which the data from the primary studies apply to the context outlined in the synthesis results [32].

**Table 2.** Characteristics of included studies.

Study	Albert et al [33]	Milberg and Strang [34]	Hull [32]	Lee et al [16]	Barlund et al [31]
Setting	Malaysia, palliative care center, Kota Kinabalu, Sabah	Sweden, hospital-based home care	United States, combined hospice program, home care	Taiwan, hospice home care, northern Taiwan	Norway, municipalities, Førde Central Hospital, Sogn og Fjordane
Aim	To explore the suffering experienced by Malaysian family members caring for patients with advanced cancer nearing end-of-life.	To describe and interpret comprehensibility and manageability experiences of informal caregivers of advanced patients with cancer in palliative home care.	To explore hospice home care experiences and perceptions of family caregivers of dying relatives.	To examine family experiences and needs when providing hospice home care to older adults with terminal cancer.	To explore factors influencing caregivers’ sense of security and facilitators for home deaths among dying patients with cancer.
Sample	First-degree relatives living with patients; primary caregivers for ≥8 hours/day	Primary caregivers at home of patients with cancer receiving hospital-based home-based palliative care	Primary home caregivers; adults living at home (with/without patient responsibilities); adults living outside home with regular care duties	Caregivers of patients with advanced cancer receiving home hospice care	Parents, children, or spouses of deceased patients with cancer
Method	Purposive sampling; semistructured in-depth interviews; thematic analysis	Maximum variation sampling; semistructured interviews; qualitative hermeneutic approach	Convenience sampling; semistructured interviews and field observation; thematic analysis	Consecutive sampling; in-depth semistructured interviews; qualitative inductive content analysis	Purposive sampling; semistructured in-depth interviews; thematic analysis
Key findings	Empathic suffering: Witnessing functional decline; fear of discomfort; receiving bad news; duties. Powerless and hopeless suffering Predictive suffering Compliance suffering: Burden of caregiving and social Barriers’ wrath: Patient-related barriers; family-related barriers; health care–related hurdles.	Comprehensibility: Congruent inner reality through open information, symbols, basic life assumptions, previous knowledge Manageability: Togetherness/isolation involving power, competence, accessibility, and support.	Confronting reality: Acute health changes, physician prognosis, treatment refusal. Surveying options: Gathering and reviewing alternatives, clarifying values, learning about hospice. Immersion: Disengaging from other tasks, shifting priorities, acquiring patient care skills. Refocusing	Hoping for cure: Concealing diagnoses from patients; expectations for prolonged life. Fluctuating emotions: Positive, negative, and difficult emotions. Accepting death: Fulfilled duties, acceptance. Perceptions of a good death: Smooth, painless, peaceful. Needs: Emotional support, information.	Personal factors Health care professionals Organizational factors

### Ethical Considerations

This meta-synthesis did not involve primary data collection, and thus ethical approval was not required. However, ethical rigor was maintained by ensuring that all included studies adhered to standard ethical guidelines, such as obtaining informed consent from participants and safeguarding participant confidentiality. Additionally, no unpublished or personally identifiable information was included in the synthesis. All sources were appropriately cited, and transparency regarding any potential conflicts of interest or funding has been maintained throughout the study.

### Positionality Statement

The reviewers overseeing the analysis and synthesis process (VL and WWST) are experienced qualitative researchers. The initial drafting, coding, and synthesis were primarily conducted by 2 researchers (XMD and KH) who have clinical backgrounds relevant to the review topic. All reviewers acknowledged that their personal characteristics, values, and beliefs, shaped by their clinical and academic experiences, could influence the synthesis process. Thus, regular discussions were held among the team to examine assumptions, challenge interpretations, and ensure a balanced and rigorous analysis of the findings to enhance reflexivity and reduce potential bias.

## Results

### Study Selection

**Figure 1.**
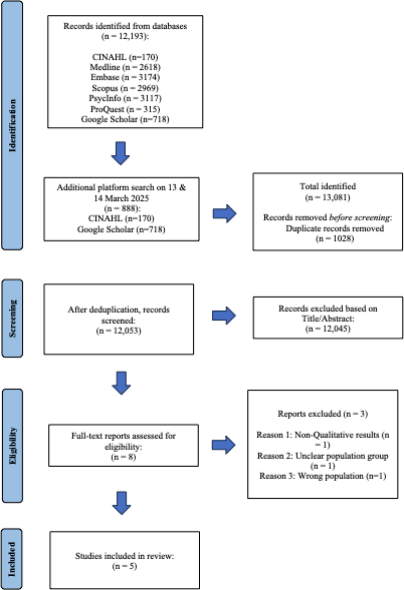
PRISMA (2020) flowchart of search results and study selection. PRISMA: Preferred Reporting Items for Systematic Reviews and Meta-Analyses.

A total of 13,081 articles were identified through our comprehensive search. This included 12,193 articles from the initial search and an additional 888 articles from the supplementary search. A total of 1028 duplicates were removed. Then, 12,053 titles and abstracts were reviewed, and 12,045 were excluded according to the eligibility criteria. Subsequently, 8 articles were read in full text, and 3 studies were excluded. Two articles were excluded due to the inability to confirm the cancer stage or to separate qualitative findings within mixed method studies, and attempts to contact the authors for verification were unsuccessful. An additional article was excluded after discussion because it predominantly contained patients’ findings, which could not be distinctly separated to focus solely on caregivers’ perspectives. Finally, 5 full-text articles met the eligibility criteria. The result of the selection process is presented in Figure 1.

### Study Characteristics

The 5 studies included in this meta-synthesis were published between 1989 and 2022, with 2 studies [31,33] published in the last 5 years. The research was conducted across diverse geographical settings: Malaysia (n=1), Sweden (n=1), the United States (n=1), Taiwan (n=1), and Norway (n=1). Study settings varied, including a palliative care center, hospital-based home care, hospice programs, and municipal or hospice-affiliated home care services.

Sample sizes across the studies ranged from small, focused groups of 12 to larger caregiver cohorts of up to 44 participants. Most studies involved between 12 and 19 participants, while one study included a substantially larger sample. One study did not report the exact number of participants. These variations reflect the diversity in study aims and sampling strategies, as well as the depth of qualitative inquiry. All participants were family caregivers of individuals with advanced cancer receiving home-based palliative care.

Sampling strategies included purposive sampling (n=3), maximum variation sampling (n=1), and consecutive or convenience sampling (n=2). Data were primarily collected through semistructured, in-depth interviews (n=5), with one study also incorporating field observation. Thematic analysis was the predominant analytical approach, although one study used a qualitative hermeneutic method and another used inductive content analysis.

Although the articles had slightly different objectives, all contributed to our understanding of the experiences, needs, and perceptions of families of patients with terminal cancer in home-based hospice care. Sampling and data collection methods varied across the studies due to population and setting differences. Four of the 5 studies were qualitative, with Hull [32] being the exception, including a quantitative portion as part of the dissertation. However, this quantitative aspect did not influence our findings, as the qualitative portion was clearly differentiated and extracted.

We noticed that one significant contributing study was notably outdated. After thorough discussion between 2 reviewers (XMD and KH), it was decided to include this study because it provided findings that remain relevant and fit well within the overall research context. Despite being published over 30 years ago in 1989 [32], the foundational concepts and findings still offer valuable insights into home-based hospice care and address aspects that are not sufficiently covered by more recent studies. The inclusion of this study ensured a comprehensive understanding of the evolution and continuity of caregiving practices. The characteristics of the included studies are presented in Table 2.

### Synthesis Findings

#### Overview

**Figure 2.**
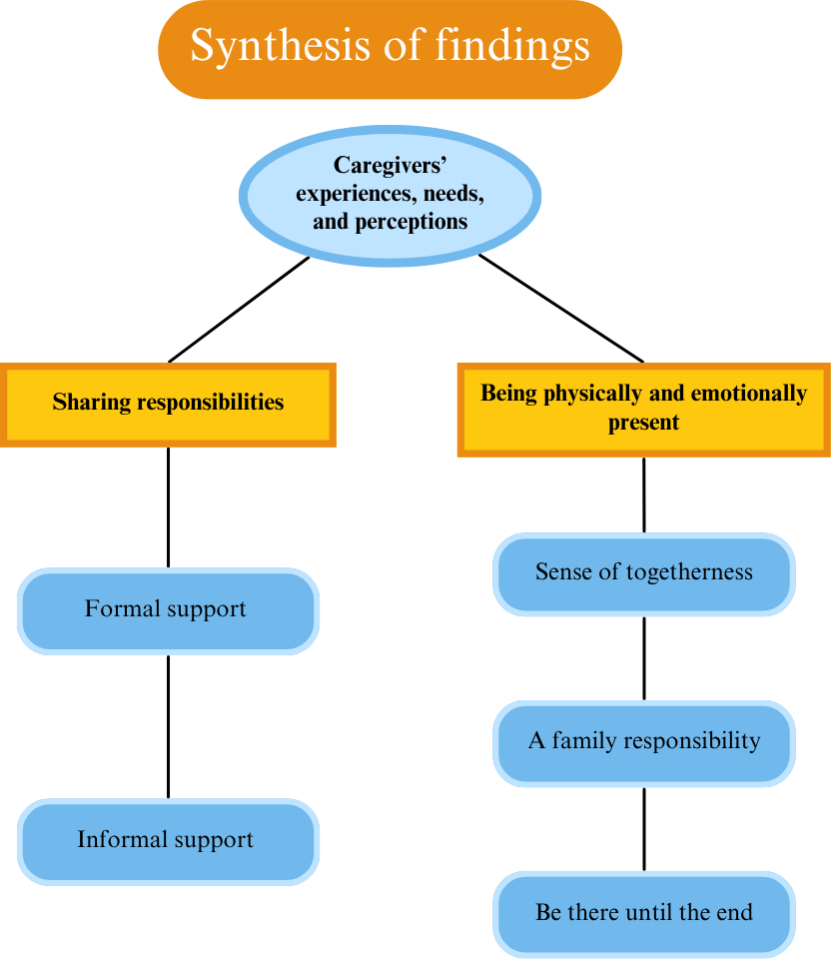
Synthesis of findings, including subthemes and main themes.

A total of 17 groups of primary codes and corresponding descriptive themes were identified. These were further synthesized into 5 overarching analytical themes, culminating in 2 central themes that represent the final synthesis. These include (1) being physically and emotionally present and (2) sharing responsibilities. The 3 subthemes that support the main theme of “being physically and emotionally present” were a sense of togetherness, family responsibility, and being there until the end. Similarly, for the main theme of “sharing responsibilities”, we synthesized the findings based on 2 subthemes: formal support and informal support (Figure 2).

Family caregivers of patients with terminal cancer made a deliberate commitment to be physically and emotionally present to care for their loved ones at home. They were motivated by the belief that they could provide a higher standard of care than what was offered in the hospital, where they observed their dying relatives being treated more as objects than as individuals with dignity. One participant expressed this sentiment: “When my dad was in the

hospital, they bathed him, fed him, and gave him his meals, and he just lay there. After these, nobody bothered him, nobody did anything more. He was nothing” [32]. At home, caregivers felt they could better meet their loved ones’ needs, taking comfort in the familiar environment and the reduced stress for both patient and caregiver. This approach offered a sense of control and personal involvement. For example, a 64-year-old daughter noted: “It didn’t take long to learn how to care for Mum. Being a mother and a wife, it was easy enough to pick up those skills, so there were no problems. I could go into nursing now (laughs); I’ve had practical experience” [32]. Similarly, a 75-year-old wife reflected on her experience: “There was no issue for me in knowing how to care for my husband. I kept him clean, the bed clean, and his pyjamas were fresh every day. It was really no problem for me” [32]. In another instance, a 35-year-old son demonstrated his capability by innovatively managing bedsores using a combination of a water mattress and an alternating mattress [32].

The 3 subthemes supported this theme: a sense of togetherness, a family responsibility, and being there until the end.

#### Sense of Togetherness

A sense of togetherness is a crucial emotional factor in home-based hospice care. Being at home made caregivers and patients feel safe and secure, knowing they were close and always within reach. One participant shared: “It was important for me to be with him... to be there all the time. And it wasn’t difficult; it felt natural. It was safe for me to be there with him” [31]. Another participant described the comfort of staying connected through a monitor: “I always had the monitor with me, even when I was out front talking to a neighbour. I kept my little walkie-talkie with me so I could always keep tabs on him” [32]. Being at home symbolized the caregivers’ commitment to staying close to the patient in the home they had built together and filled with memories, despite the good or bad times. One participant poignantly remarked:

This is our home, and this is where she should be as long as she lives. She’s receiving better care here than she would anywhere else, and as long as I’m here, she’ll have that care as long as it’s needed. This is where she belongs. This is her life’s work. We raised our family here for 38 years. I lived through the good years; now I want to live through the bad. What’s one bad year out of 47? [32].

#### A Family Responsibility

From a cultural perspective, caregiving is a natural extension of one’s commitment to loved ones and a moral obligation. For many children caregivers, taking on the responsibility of caring for their parents with terminal illness at home is deeply rooted in cultural and ethical values, often viewed as a way to repay their parents. In this cultural context, there is a strong belief in managing care independently rather than relying on nursing homes to express filial duty. This sentiment is reflected in the words of a daughter from Taiwan: “Because my father’s condition was so bad, it made my heart ache to watch him suffering from pain...I wanted to care for my father on my own and did not need anyone else to bear my responsibility” [16]. Another participant echoed this sense of responsibility: “The most important thing for me to learn is that you have to give yourself an opportunity to take care of your sick parents who have taken care of you since you were a child...I was really proud of becoming familiar with the skills of care” [16]. This highlights the emotional significance of caregiving, which carries a deep sense of responsibility. Many caregivers, particularly those who were the sons or daughters of patients with cancer, believed they had fulfilled their filial duties and wanted their loved ones to have a natural death and a proper funeral. As one son expressed: “We will not have regrets if our father dies tomorrow...We believe we demonstrated the value of filial piety as much as we could by taking good care of him at home without sending him to a nursing home...we knew he wanted to die at home” [16].

#### Be There Until the End

However, families often face significant emotional challenges when discussing topics related to death or illness while striving to be emotionally present for their loved ones. In Sweden, being present was crucial, even when it was difficult to discuss such matters. One participant shared: “She [mother] is talking a bit too much about the funeral and such things. I find it quite burdensome. But it is good for her, so we talk” [34]. Some family members chose to hide terminal diagnoses from their loved ones in an attempt to protect them from losing hope, believing that revealing the full extent of the illness might cause them to give up on life [16]. In Malaysia, caregivers also expressed a strong desire to remain with their loved ones and provide care until the end, keeping them happy and shielding them from their emotions. One caregiver shared: “I do not want him to see me crying. I want him to be happy. I know that I am the only one next to him. If I were there with a sad feeling...I think it might make him sad” [33]. The complexity of providing compassionate care while managing one’s emotional burdens is evident in these experiences, underscoring families’ challenges in navigating the end-of-life journey.

#### Sharing Responsibilities

Sharing responsibilities in caregiving can involve the patient, family, friends, home palliative care teams, and other community organizations. This collaborative approach helped family caregivers feel less isolated when managing challenging situations. Throughout the caregiving process, these groups play a vital role in supporting family caregivers of patients with terminal cancer. It is essential to recognize that family caregivers often require various types of support. This theme was further supported by 2 subthemes: support from a palliative home-based care team and support from others.

#### Formal Support

The involvement of a palliative home care team can help caregivers perceive caregiving as more manageable [34], boosting their confidence in providing care for their loved ones at home [16]. A Swedish participant shared a poignant experience:

My husband was going to be discharged from the hospital [with a percutaneous endoscopic gastrostomy], but I said I can’t take care of such things. But they said it was very easy to learn how to use it. ‘No,’ I said. ‘I can’t take that responsibility’...Then the dietician came and said, ‘It is so easy.’ I felt I was going to be ill because I could not do this. (Sighs) And later on that afternoon, I had diarrhoea. I was not feeling well and was terribly worried...Then the palliative doctor came, and he was almost like an angel. He presented all the things the palliative home care team could offer. And then I felt that this was a support for us [34].

The need for qualified health care professionals is often emphasized, as they are the support personnel to whom caregivers can express their concerns, thoughts, and worries. This interaction gave caregivers confidence and security, reassuring them that they appropriately fulfilled their caregiving role. Caregivers welcome palliative care nurses or coordinators, as one participant reported:

“It felt safe and secure for us to know that they were visiting us (ie, home nursing care)” [31].

Some caregivers acknowledged that having palliative home care made it easier to fulfill their wish to care for patients at home during the final stage of life, facilitating a dignified end-of-life experience. As one son reflected:

“I thought my mother’s death was a good death because she passed away without pain or any distressing symptoms from cancer. It was really important for us, and we appreciated what the hospice home-based care team did for us” [16].

#### Informal Support

Other family members, relatives, and friends play a crucial role in supporting family caregivers who provide care for patients with terminal cancer. In families with children, caregivers were glad to share their responsibility with their offspring, viewing it as an expression of filial piety. One participant said:

“I was glad to see my son helping me care for his dad...I thought that if I’m sick someday he would care for me like now and our relationship gave me the energy to care for my husband” [16].

Managing the financial responsibilities of caregiving often necessitates sharing the burden with other family members or government organizations. The emotional and physical toll of caregiving can be overwhelming, as one participant poignantly expressed:

“I declined the offer of attendance allowance; I wanted another sister to do this. I didn’t want to be alone in this [ie, follow the patient in the last phase]. I wanted more people to be involved because I had... (Sighs) I had been doing this alone for so long” [31].

This highlights the deep need for collective involvement, especially in the final stages of care, which can be particularly draining when borne alone.

### Quality Appraisal of Included Studies

The methodological quality of the included studies was assessed using the CASP Qualitative Checklist. All 5 included studies demonstrated generally high methodological quality, with scores ranging from 29 to 30 points out of a possible maximum score of 30 points (Table 3). All studies clearly stated their research aims, adopted appropriate qualitative methodologies, applied suitable research designs, and used adequate recruitment strategies and data collection methods. Four studies clearly considered ethical issues, while 2 lacked explicit discussion regarding the relationship between researchers and participants, introducing minor ambiguity. Nevertheless, all included studies clearly articulated their findings and demonstrated valuable contributions to the topic. A detailed summary of the quality appraisal results is presented in Table 3.

**Table 3.** Quality appraisal of the included studies using the Critical Appraisal Skills Programme (2019).

	Albert et al, 2022 [33]	Milberg and Strang, 2004 [34]	Hull, 1989 [32]	Lee et al, 2014 [16]	Barlund et al, 2021 [31]
Was there a clear statement of the aims of the research?	+	+	+	+	+
Is a qualitative methodology appropriate?	+	+	+	+	+
Was the research design appropriate to address the aims of the research?	+	+	+	+	+
Was the recruitment strategy appropriate to the aims of the research?	+	+	+	+	+
Was the data collected in a way that addressed the research issue?	+	+	+	+	+
Has the relationship between researcher and participants been adequately considered?	+	+/–	+	+	+/–
Have ethical issues been taken into consideration?	+	+	+/–	+/–	+
Was the data analysis sufficiently rigorous?	+	+	+	+	+
Is there a clear statement of findings?	+	+	+	+	+
Is the research valuable?	+	+	+	+	+
Total points	30	29	29	29	29

### Confidence of Evidence

Two major themes emerged from the thematic synthesis: (1) being physically and emotionally present and (2) sharing responsibilities. Both themes were assessed with high confidence according to the GRADE-CERQual framework (Table 1). The theme “being physically and emotionally present” had no or very minor concerns regarding methodological limitations and coherence, minor concerns related to data adequacy, and minor concerns regarding relevance due to the partial inclusion of studies addressing home- and hospital-based settings. Similarly, the theme “sharing responsibilities” had no or minor problems related to methodological limitations, coherence, and adequacy, with minor concerns regarding relevance, given the partial relevance of 2 included studies that covered broader contexts beyond home-based care. These results demonstrate robust qualitative evidence reflecting the experiences, perceptions, and needs of family caregivers in home-based hospice care for patients with terminal cancer.

## Discussion

### Principal Findings

This review synthesized findings from 5 qualitative studies exploring family caregiver experiences, perceptions, and needs in home-based hospice care for patients with terminal cancer. Two prominent themes emerged: (1) being physically and emotionally present, highlighting caregivers’ dedication and the emotional complexities involved in caregiving, and (2) sharing responsibilities, demonstrating the importance of formal and informal support systems. These themes reflect the complex emotional and practical challenges caregivers encounter while striving to provide high-quality care aligned with their loved ones’ wishes.

### Comparison to Prior Work

Our findings align with previous research highlighting family caregivers’ essential role in delivering compassionate and dignified end-of-life care [40]. The emotional dedication caregivers demonstrate, often deeply embedded in cultural expectations such as filial piety, confirms existing literature emphasizing the profoundly personal nature of caregiving. However, this synthesis also underscores specific challenges faced by caregivers, including managing difficult conversations about death, balancing caregiving responsibilities, and navigating cultural norms, consistent with prior research identifying caregiver stress and potential burnout risks.

These findings align with established social support theories, confirming that emotional, informational, and instrumental support from formal (health care professionals) and informal networks (family and friends) substantially alleviates caregiving stress [41]. Nonetheless, our analysis revealed a significant gap in integrating these support systems, leaving caregivers vulnerable to isolation and overwhelm.

### Strengths and Limitations

The strength of this review includes the rigorous methodological approach followed, adherence to PRISMA guidelines, quality appraisal using the CASP checklist, and confidence assessment using GRADE-CERQual. Including diverse cultural contexts from Asian and Western countries further enhances the generalizability and applicability of our findings.

However, this review has several limitations. First, limiting the search to studies published in English may have restricted the inclusion of research conducted in non–English-speaking regions, thereby reducing the diversity in cultural contexts and settings across the included studies and potentially affecting the generalizability of the findings [42]. Second, despite the growing global reliance on family caregivers in home-based hospice care [43], this review identified only 5 eligible studies, with just 2 published in the past 5 years. This limited number may reflect the specificity of our inclusion criteria, which focused solely on caregivers of patients with terminal cancer receiving home-based palliative care. Many existing end-of-life care studies include a broader population with varied diagnoses and may not isolate the unique caregiving experience related to cancer. This scarcity highlights a critical gap and the need for more focused, culturally diverse qualitative research in this area [44]. Third, the retrospective nature of some studies introduced a potential recall bias, affecting the accuracy of reported experiences. This could be addressed in future research through prospective study designs. Fourth, our review’s reliance on specific databases potentially limited the comprehensiveness of identified literature, mainly gray literature. Future studies should broaden database searches and proactively include unpublished literature to enhance comprehensiveness. Lastly, including older literature (more than 5 years old) may limit the review’s alignment with the most current evidence. However, older studies were included due to their foundational insights, which remain relevant to the current practice context. Future research should emphasize more recent publications to align closely with contemporary practices and emerging evidence.

### Future Directions

The findings indicate several avenues for future research and practice. There is a clear need to develop and evaluate culturally sensitive interventions tailored to both formal and informal caregiver support needs. Initiatives should aim to bridge existing gaps in caregiver support through integrated services that alleviate isolation and promote emotional and practical caregiving capacities. Structured educational programs and support groups designed to improve communication around end-of-life topics could also substantially enhance caregiver experiences.

### Conclusion

Home-based hospice care has a significant impact on patients, but the experience of family members who support them remains neglected in literature and daily practices. The results highlight both the emotional rewards and daunting challenges caregivers encounter and point to the need for systemic, culturally competent strategies to support this population. By bridging the gaps within formal and informal support systems and encouraging open communication, these health care providers can enable caregivers to maneuver their roles effectively. These findings present critical implications for the delivery of hospice care as the world learns to provide holistic, compassionate care, without fail, for patients and their families. Future research should investigate diverse caregiver experiences and further inform the refinement of focused interventions to improve home-based hospice care.

In conclusion, this review highlights the critical role of caregivers in home-based hospice care for patients with terminal cancer, emphasizing the need for culturally competent, comprehensive caregiver support strategies. Addressing identified gaps can significantly improve caregivers’ experiences and ultimately enhance the quality of hospice care services delivered at home.

## Supplementary material

10.2196/71596 Multimedia Appendix 1 Inclusion and exclusion criteria.

10.2196/71596 Multimedia Appendix 2 Full search strategy.

10.2196/71596 Checklist 1 PRISMA checklist. PRISMA: Preferred Reporting Items for Systematic Reviews and Meta-Analyses.

## Abbreviations

CASP: Critical Appraisal Skills Programme
GRADE-CERQual: Grading of Recommendations Assessment, Development, and Evaluation–Confidence in the Evidence from Reviews of Qualitative Research
MeSH: Medical Subject Headings
PICo: Population, Interest, Context
PRISMA: Preferred Reporting Items for Systematic Reviews and Meta-Analyses
PROSPERO: Prospective Register of Systematic Reviews
