# Supplementary material for: Family Experiences, Needs, and Perceptions in Home-Based Hospice Care for Patients With Terminal Cancer: Meta-Synthesis and Systematic Review
Source: JMIR Cancer. 2025 Jun 19;11:e71596. doi: 10.2196/71596 (PMC12202240; doi:10.2196/71596)
Supplement: Multimedia Appendix 1 [file cancer-v11-e71596-s001.docx]

| **APPENDIX B - INCLUSION & EXCLUSION CRITERIA (PICo)** | | |  |
| --- | --- | --- | --- |
|  | ***P****- Population* | ***I****-  Interest* | ***Co****- Context* |
| ***INCLUSION CRITERIA*** | Adult families of advanced cancer patients under home-based hospice care with physical home visits by healthcare team | Experience, perception, and needs under home-based hospice care | Home-based hospice services with physical home visits.  Primary studies employing qualitative or mixed methods designs, with qualitative components distinctly extractable.  Published and unpublished studies in English |
| ***EXCLUSION CRITERIA*** | Studies focusing on patients themselves only; Healthcare providers | N.A. | Non-home-based hospice services (e.g., inpatient hospice, nursing home care) and telemedicine-only visits.  Studies that are not in English  Mixed methods studies that are not able to extract the data collected and analysed using qualitative methods. |
