# Supplementary material for: Family Experiences, Needs, and Perceptions in Home-Based Hospice Care for Patients With Terminal Cancer: Meta-Synthesis and Systematic Review
Source: JMIR Cancer. 2025 Jun 19;11:e71596. doi: 10.2196/71596 (PMC12202240; doi:10.2196/71596)
Supplement: Multimedia Appendix 2 [file cancer-v11-e71596-s002.docx]

**APPENDIX 3: FULL SEARCH STRATEGY**

| Database: *Medline (PubMed)*  Date of Search:  **13 ‎September ‎2023, 13:12**  No. of Results: 2618  Limits/Filters applied: English (language); mixed-method studies; qualitative studies. |
| --- |
| **Search Strategy:**  ***P (Population)***  "neoplasms"[MeSH Terms] OR "neoplasms, multiple primary"[MeSH Terms] OR "carcinoma"[MeSH Terms] OR "neoplasm, residual"[MeSH Terms] OR "neoplasm recurrence, local"[MeSH Terms] OR "neoplasm metastasis"[MeSH Terms] OR "neoplasms by site"[MeSH Terms] OR "mixed tumor, malignant"[MeSH Terms] OR "neoplasms, second primary"[MeSH Terms] OR ("cancer"[Title/Abstract] OR "malignancy"[Title/Abstract] OR "tumor"[Title/Abstract] OR "tumour"[Title/Abstract]  ***I (Interest)***  "palliative care"[MeSH Terms] OR "hospice care"[MeSH Terms] OR "hospices"[MeSH Terms] OR "home care services"[MeSH Terms] OR "home nursing"[MeSH Terms] OR "palliative medicine"[MeSH Terms] OR "terminal care"[MeSH Terms] OR "hospice and palliative care nursing"[MeSH Terms] OR ("end of life care"[Title/Abstract] OR "palliative"[Title/Abstract] OR "hospice"[Title/Abstract] OR "terminal care"[Title/Abstract] OR "hospice care"[Title/Abstract] OR cancer-care"[Title/Abstract]  ***Co (Context)***  "home nursing"[Title/Abstract] OR "outreach palliative"[Title/Abstract] OR "home based care"[Title/Abstract] OR "home based care"[Title/Abstract] OR "cancer-care"[Title/Abstract] |
|  |
| Database: *Embase*  Date of Search: **13 September 2023, 13:15.**  No. of Results: 3174  Limits/Filters applied: English (language); mixed-method studies; qualitative studies. |
| Search Strategy:  ***P (Population)***  'cancer':ab,ti OR 'cancers':ab,ti OR 'carcinoma':ab,ti OR 'carcinoma'/exp OR 'metastasis':ab,ti OR 'metastasis'/exp OR 'neoplasm':ab,ti OR 'neoplasm'/exp OR 'malignant neoplasm':ab,ti OR 'malignant neoplasm'/exp OR 'mixed tumor'/exp  ***I (Interest)***  'palliative nursing':ab,ti OR 'palliative nursing'/exp OR 'palliative therapy':ab,ti OR 'palliative therapy'/exp OR 'terminal care':ab,ti OR 'terminal care'/exp OR 'visiting nursing service':ab,ti OR 'visiting nursing service'/exp OR 'hospice nursing'/exp OR 'hospice care'/exp OR 'home care':ab,ti OR 'home care'/exp  ***Co (Context)***  experience*:ab,ti OR perception*:ab,ti OR need*:ab,ti OR experience* |
|  |
| Database: *SCOPUS*  Date of Search: **13 September 2023, 13:26.**  No. of Results: 2969  Limits/Filters applied: English (language); mixed-method studies; qualitative studies. |
| **Search Strategy**:  ***P (Population)***  metastasis* OR neoplasm* OR cancer* OR carcinoma*  ***I (Interest)***  "palliative* care*" OR home AND hospice AND care OR "terminal* care*" OR "home* nursing*" OR "outreach* palliative*" OR "home-based* care*" OR "hospice* care*"  ***Co (Context)***  experience* OR need* OR perception* OR perspective* OR view* OR attitude* OR feeling* |
|  |
| Database: PsycInfo  Date of Search: ‎**13 September 2023, ‏‎13:26**  No. of Results: 3117  Limits/Filters applied: English (language); mixed-method studies; qualitative studies. |
| **Search Strategy:**  ***P (Population)***  exp Neoplasms/ or cancer.mp. or cancers.mp. or carcinoma.mp. or metastasis.mp. or exp Metastasis or malignancy.mp. or tumor.mp. or tumors.mp.  ***I (Interest)***  exp Palliative Care/ or exp Home Care/ or palliative nursing.mp. or exp Hospice/ or terminal care.mp. or hospice care.mp. or home hospice.mp. or exp Home Visiting Programs/ or visiting nursing service.mp. or hospice nursing.mp. or patient centered care/  ***Co (Context)***  experience.mp. or perception.mp. or exp Perception/ or needs/ or health service needs/ or psychological needs/ or need satisfaction/ or exp Life Experiences/ or exp Attitudes/ or attitude.mp. |
|  |
| Database: ProQUEST  Date of Search: **13 September 2023, 13:45.**  No. of Results: 315  Limits/Filters applied: English (language); mixed-method studies; qualitative studies; ProQuest Dissertations and Theses; full-text only. |
| **Search Strategy:**  ***P (Population)***  (abstract(cancer OR cancers OR metastasis OR malignancies OR malignancy OR neoplasm) OR title(cancer OR cancers OR metastasis OR malignancies OR malignancy OR neoplasm))  ***I (Interest)***  (abstract(palliative care OR home hospice care OR home care OR outreached palliative OR home-based care) OR title(palliative care OR home hospice care OR home care OR outreached palliative OR home-based care))  ***Co (Context)***  (abstract(experience* OR need* OR perception* OR attitude* OR perspective* OR view*) OR title(experience* OR need* OR perception* OR attitude* OR perspective* OR view*) OR subject(experience* OR need* OR perception* OR attitude* OR perspective* OR view*) |
|  |
| Database: CINAHL  Date of Search: **13 March 2025, 19:00.**  No. of Results: 170  Limits/Filters applied: Publication Date: 2018–2025; English only. |
| (TI (cancer OR cancers OR metastasis OR malignancies OR malignancy OR neoplasm) OR AB (cancer OR cancers OR metastasis OR malignancies OR malignancy OR neoplasm) OR MH "Neoplasms+")  AND  (TI ("home hospice care" OR "home-based hospice care" OR "home palliative care" OR "home-based palliative care" OR "home care" OR "outreach palliative care") OR  AB ("home hospice care" OR "home-based hospice care" OR "home palliative care" OR "home-based palliative care" OR "home care" OR "outreach palliative care") OR  MH ("Hospice Care+" OR "Home Health Care" OR "Palliative Care"))  AND  (TI ("family caregiver*" OR caregiver* OR carer* OR relative* OR informal caregiver*) OR  AB ("family caregiver" OR "informal caregiver" OR caregiver* OR carer*) OR  MH ("Caregivers" OR "Family Caregivers" OR "Family"))  AND  (MH "Qualitative Studies+" OR MH "Quantitative Studies" OR MH "Mixed Methods Studies" OR MH "Interviews+" OR MH "Focus Groups" OR MH "Narratives" OR MH "Life Experiences+" OR MH "Surveys and Questionnaires+" OR MH "Questionnaires+" OR MH "Focus Groups" OR TI ("qualitative" OR "quantitative" OR "mixed method*" OR "mixed-method*") OR AB ("qualitative" OR "quantitative" OR "mixed methods"))  AND  (TI (experience OR perception OR attitude OR need OR view OR perspective OR feeling) OR  AB (experience OR perception OR attitude OR need OR view OR perspective OR feeling) OR  MH ("Attitude" OR "Perception" OR "Life Experiences+" OR "Needs Assessment"))  LIMIT TO (Full Text) AND (English)  AND  Publication Date: 2018–2025 |
|  |
| Database: Google Scholar  Date of Search: **14 March 2025, 09:00.**  No. of Results: 718  Limits/Filters applied: Publication Date: 2018–2025; English only. |
| **Search Strategy:**  ***P (Population)***  (intitle:cancer OR intitle:cancers OR intitle:metastasis OR intitle:malignancies OR intitle:neoplasm OR intext:cancer OR intext:malignancies OR intext:neoplasm)  AND  (intitle:caregiver OR intitle:"family caregiver" OR intitle:carer OR intitle:"informal caregiver" OR intitle:"primary caregiver" OR intext:caregiver OR intext:"family caregiver" OR intext:carer OR intext:"informal caregiver" OR intext:"primary caregiver")  ***I (Interest)***  (intitle:"home hospice" OR intitle:"home-based hospice" OR intitle:"home palliative care" OR intitle:"home-based palliative care" OR intitle:"home care" OR intitle:"outreach palliative" OR intext:"home hospice" OR intext:"home-based hospice" OR intext:"home palliative care" OR intext:"home-based palliative care" OR intext:"home care" OR intext:"outreach palliative")  ***Co (Context)***  (intitle:qualitative OR intitle:quantitative OR intitle:"mixed method" OR intitle:"mixed methods" OR intext:qualitative OR intext:quantitative OR intext:"mixed methods")  AND  (intitle:experience OR intitle:perception OR intitle:attitude OR intitle:need OR intitle:view OR intext:experience OR intext:perception OR intext:attitude OR intext:view OR intext:needs) |

Footnote: * is a truncation symbol to retrieve terms with a common root within EMBASE, SCOPUS and MEDLINE.
